# The INTER-ACT E-Health Supported Lifestyle Intervention Improves Postpartum Food Intake and Eating Behavior, but Not Physical Activity and Sedentary Behavior—A Randomized Controlled Trial

**DOI:** 10.3390/nu13041287

**Published:** 2021-04-14

**Authors:** Margriet Bijlholt, Lieveke Ameye, Hanne Van Uytsel, Roland Devlieger, Annick Bogaerts

**Affiliations:** 1 Centre for Research and Innovation in Care (CRIC), Faculty of Medicine and Health Sciences, University of Antwerp, 2610 Antwerp, Belgium; margriet.bijlholt@uantwerpen.be; 2 Research Unit Woman and Child, Department of Development and Regeneration, KU Leuven, 3000 Leuven, Belgium; lieveke.ameye@kuleuven.be (L.A.); hanne.vanuytsel@kuleuven.be (H.V.U.); roland.devlieger@uzleuven.be (R.D.); 3 Department of Obstetrics and Gynecology, University Hospital Leuven, 3000 Leuven, Belgium; 4 Faculty of Health, University of Plymouth, Devon PL4 8AA, UK

**Keywords:** obesity, gestational weight gain, postpartum, postnatal, interpregnancy

## Abstract

Unhealthy postpartum lifestyle is related to long-term adverse psychological, metabolic and cardiovascular health outcomes as well as to complications in the next pregnancy. Especially women with preceding excessive gestational weight gain are at risk. This paper aims to evaluate the effect of the postpartum phase of the INTER-ACT randomized controlled trial (RCT) on food intake, eating behavior, physical activity and sedentary time at the end of the intervention (six months postpartum) and at six-months follow-up (12 months postpartum). The study population comprised women with excessive gestational weight gain in the preceding pregnancy. The lifestyle intervention combined a smartphone application with four face-to-face coaching sessions between six weeks and six months postpartum. After the intervention, restrained eating score was 1 point higher (95% CI 0.5, 1.5; *p* < 0.001), uncontrolled eating score was 1 point lower (95% CI −1.9, −0.2; *p* = 0.02) and energy intake was 69 kcal lower (95% CI −123, −15; *p* = 0.01) in the intervention group compared to the control group. The differences were no longer statistically significant at follow-up. No significant effects on emotional eating, physical activity and sedentary behavior were found. In conclusion, the postpartum phase of the INTER-ACT RCT was effective in improving nutrition-related outcomes, however, these improvements could not be sustained at follow-up. ClinicalTrials.gov identifier: NCT02989142.

## 1. Introduction

Unhealthy lifestyle patterns in the postpartum period are related to adverse psychological, metabolic, and cardiovascular health outcomes in later life for both the mother as well as the child [1,2,3,4,5]. Postpartum weight retention is a potential consequence of unfavorable lifestyle and, in turn, contributes to an increased risk of adverse pregnancy outcomes in a subsequent pregnancy [2,6,7,8]. Especially women who had excessive weight gain in the preceding pregnancy are at increased risk of postpartum weight retention and therefore of complications in the next pregnancy [9]. Excessive gestational weight gain is defined by the National Academy of Medicine as >9 kg gestational weight gain in mothers with an obese BMI at start pregnancy, >11.5 kg in mothers with overweight at start, and >16 kg in mothers with a normal body–mass index (BMI) at the start of pregnancy [10]. Excessive gestational weight gain is a common condition with a prevalence of approximately 35% in Belgian pregnant women and up to 50% in the United States [9,11,12].

Several postpartum lifestyle interventions have been described in the literature, the majority primarily aiming to reduce postpartum weight retention through lifestyle adaptations [13]. Short, intensive interventions that incorporate a diet only or diet and physical activity component seem most effective in reducing postpartum weight retention. Only a minority of studies incorporate e-health components into their interventions, even though this can be an advantageous extension to face-to-face interventions [14]. Besides, it is uncommon in existing interventions to perceive the postpartum period as inter-pregnancy period and intend to use this period as an opportunity to prepare for a healthy preconception phase. Furthermore, none of the studies specifically focus on women who had excessive gestational weight gain in their preceding pregnancy, although special attention on this high-risk group is required [13].

The INTER-ACT randomized controlled trial (RCT) aims to reduce pregnancy complications through an e-health and face-to-face combined lifestyle intervention. The intervention commences as early as the postpartum period of a preceding pregnancy, which is necessary to timely reach at-risk women and prepare for a healthy preconception period of the next pregnancy. The study specifically targets women who had an excessive gestational weight gain in the preceding pregnancy, since these women are at increased risk of pregnancy- and birth related complications in the next pregnancy. The current paper aims to evaluate whether the postpartum phase of the INTER-ACT RCT is effective in improving lifestyle measures, including food intake, eating behavior, physical activity and sedentary time, at the end of the intervention and at six months follow-up. We hypothesize that the intervention results in a lower energy intake, improved eating behavior, increased physical activity and decreased sedentary time.

## 2. Materials and Methods

The methodology of the INTER-ACT RCT has been previously described in detail elsewhere [15]. In summary, the intervention group received the postpartum intervention which took place at week 6, 8, 12 and 6 months after delivery. The only difference between intervention and control group in terms of intervention was that the intervention group received the four coaching sessions and the smartphone application, which was not offered to the control group. Baseline and follow-up measurements took place at same time points in both randomized arms, that is, week 6 (baseline measurement), month 6 and month 12 after delivery. The study was conducted according to the guidelines of the Declaration of Helsinki and was approved on 9 March 2017 by the Ethics Committees of the participating hospitals (protocol code B322201730956). The study was pre-registered on ClinicalTrials.gov (NCT02989142).

### 2.1. Participants

Participants were recruited in six hospitals in the region of Flanders, Belgium: University Hospital Leuven, University Hospital Antwerp, Gasthuiszusters Hospitals Antwerp, Jessa Hospital in Hasselt, Hospital Oost-Limburg in Genk, and Sint-Franciscus Hospital in Heusden-Zolder. Between May 2017 and April 2019, research midwives approached potential participants two to three days after delivery in the hospital. They recruited women with excessive gestational weight gain as defined by the National Academy of Medicine [10]. Women aged 18 or older with a sufficient command of the Dutch language were eligible for enrolment. Women who gave birth to twins, had a stillbirth, previously had bariatric surgery or planned one, or had a chronic or psychiatric disorder were ineligible. All participants provided informed consent upon recruitment.

Due to small numbers, women with pre-pregnancy underweight were excluded from the current analyses (*n* = 16). Women who got pregnant again within the first year postpartum were excluded from the current analyses from that time point onwards (*n* = 9 excluded from end of intervention and *n* = 77 excluded from follow-up analyses).

### 2.2. Randomization

Participants were randomized into the intervention group or control group with an allocation ratio 1:1. The randomization algorithm available in the electronic case report form (eCRF) Castor was used—block randomization with block sizes 4, 6 and 8. At every block generation moment, one of the three block sizes was randomly selected. When a record was randomized, the allocation was randomly selected from the current block in use. The randomization was stratified by hospital. Participants were enrolled by research midwives, and randomized was performed by the biostatistician within the 1st week postpartum. Due to the nature of the intervention, participants and study personnel could not be blinded for randomization. All caregivers involved in the perinatal care path were blinded for the allocation of a specific woman.

### 2.3. Intervention

The lifestyle intervention focused on nutrition, eating behavior, physical activity, sedentary behavior and mental wellbeing delivered by a combination of face-to-face coaching and purpose-designed smartphone application [16].

Coaching sessions took place at 6 weeks, 8 weeks, 12 weeks and 6 months after delivery. For organizational reasons, a range of two weeks before to four weeks after these time points was allowed. A second intervention phase was initiated when participants conceived again, however, the current paper only focuses on the postpartum phase of the intervention. A manual was developed in order to enhance similar implementation of the coaching sessions between the different coaches and coaches received a three-day training session. Motivational interviewing, behavioral change techniques, goal setting, action planning and reinforcement were implemented in the coaching sessions. As to the preference of the participants, coaching sessions took place individually or in small groups of a maximum of three participants.

The smartphone application was installed on the participant’s phone at six weeks postpartum and supported the coaching sessions throughout the intervention. Participants who did not own a smartphone received one. Motivational messages and informational tips were sent through the application and participants could set and monitor their own lifestyle goals. Furthermore, the application connected with an activity tracker (Withings GO) and weighing scale (Withings Body+, Withings, Issy-les-Moulineaux, France) that were provided to the intervention group participants to stimulate self-monitoring of physical activity and body weight. The development process and field evaluation of the smartphone application are previously described in detail elsewhere [16].

### 2.4. Data Collection

All data were captured in the eCRF Castor. Medical record data was collected upon recruitment by the research midwives. Data on lifestyle behaviors were collected at six weeks postpartum (baseline), six months postpartum (end of intervention) and twelve months postpartum (six-month follow-up) by means of an online questionnaire emailed to the participants. Sociodemographic information was only collected once at baseline.

Data on frequencies and portions of 48 food groups consumed in the past month were collected using a validated food frequency questionnaire [17]. Average daily energy intake was calculated in kilocalories.

Restrained, uncontrolled and emotional eating behaviors were assessed using the Three Factor Eating Questionnaire Revised 18-item version [18]. Restrained eating comprised of six items, uncontrolled eating of nine items, and emotional eating of three items. The items were scored on a four-point Likert scale. Consequently, the possible range of scores were 6–24 for restrained, 9–36 for uncontrolled and 3–12 for emotional eating.

The International Physical Activity Questionnaire was used to assess physical activity in metabolic equivalent of task-minutes (MET-minutes) per week and sedentary time in minutes per day [19].

### 2.5. Data Analysis

Data were analyzed for the body mass index (BMI) groups together as well as stratified for normal weight (18.5–24.9 kg/m^2^), overweight (25.0–29.9 kg/m^2^) and obesity (≥30 kg/m^2^). To assess the effect of the INTER-ACT intervention on eating behavior, food intake, physical activity and sedentary time, univariate analyses were performed using an independent samples *t*-test for parametric outcome variables and Mann-Whitney U test for non-parametric outcome variables. Multiple linear regression analyses were carried out to adjust for method of conception and kilograms exceeding guidelines of gestational weight gain, which differed between control and intervention group at baseline despite randomization. Results were expressed as mean differences and 95% confidence intervals. MET-minutes for physical activity was non-parametric and was therefore first log-transformed to meet assumptions of multiple linear regression. Emotional eating scores were strongly skewed and transformation of the data did not improve this. Therefore, emotional eating scores were dichotomized into low and high emotional eating based on the median score, and logistic regression was performed to test differences between control group and intervention group. All analyses were performed using SPSS version 26 and SAS version 9.4.

## 3. Results

**Figure 1:**
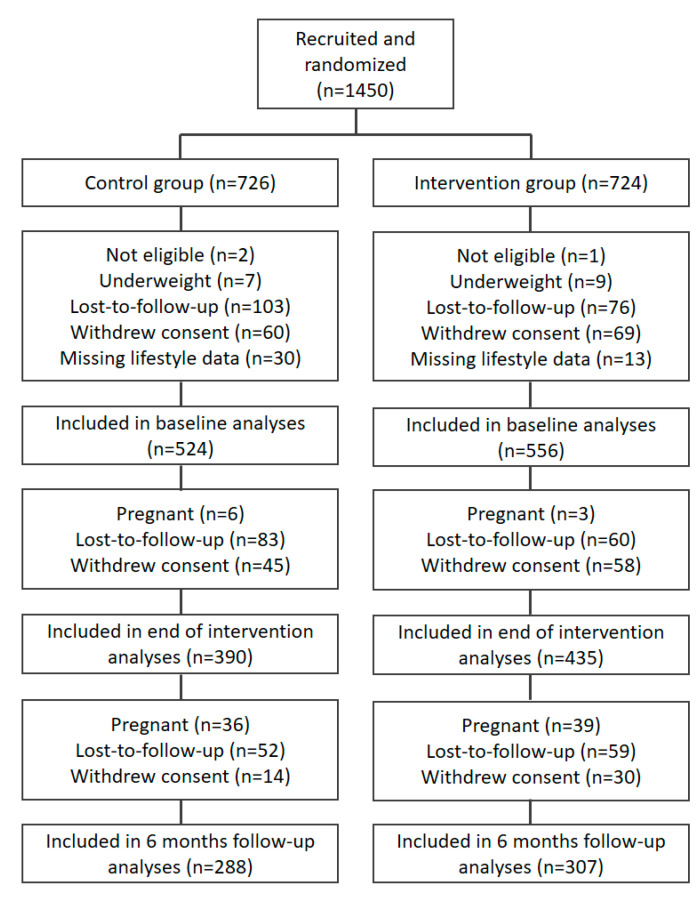
Flow chart of participant follow-up.

The participant flow is displayed in Figure 1. A total of 1450 participants were recruited, of which 726 were randomized into the control group and 724 into the intervention group. At baseline, 524 control group participants and 556 intervention group participants were included in analyses, at end of intervention respectively 390 and 435, and, at six months follow-up, respectively, 288 and 307 participants (Figure 1).

Participants were on average 31.3 years old and no statistical difference was shown with pre-pregnancy BMI category nor with drop-out over time. Half of participants were primiparous (54.8%) and gave birth to a boy (53.4%).

Almost half of the participants had a normal pre-pregnancy BMI (48.6%), 36.0% had a pre-pregnancy BMI in the overweight range, and 15.4% in the obese range (Table 1). Despite 1:1 randomization, some significant differences existed between control and intervention group at baseline. More women conceived their pregnancy spontaneously in the control group (92.4%) compared to the intervention group (88.3 percent, *p* = 0.03). Among women with a normal pre-pregnancy BMI, median gestational weight gain was slightly higher in the control group (19 kg, Q1–Q3 17–21 kg) compared to the intervention group (18.2 kg, Q1–Q3 17–20 kg, *p* = 0.04) (Table 1). To take these differences into account, the variables were included as covariates in the multivariate regression analyses. All statistically significant differences that were found between control and intervention group in the univariate analyses remained significant after adjusting for kilograms of excessive weight gain and method of conception in the multivariate analyses, indicating that these covariates did not influence the results (Table 2).

### 3.1. Eating Behavior and Food Intake

At the end of the intervention (6 months postpartum), restrained eating score was one point higher (95% CI 0.5, 1.5; *p* < 0.001), uncontrolled eating score was one point lower (95% CI −1.9, −0.2; *p* = 0.02) and energy intake was 69 kcal lower (95% CI −123, −15; *p =* 0.01) in the intervention group compared to the control group. Stratification of the BMI groups showed that significant differences between control and intervention group in restrained eating and uncontrolled eating were specifically found in women with normal pre-pregnancy weight (respectively, 1.4 points higher; 95% CI 0.6, 2.1; *p* < 0.001 and 1.6 points lower; 95% CI −2.7, −0.5; *p =* 0.004), and differences in energy intake were specifically seen in participants with an obese pre-pregnancy BMI (174 kcal lower; 95% CI −314, −35; *p =* 0.01). A similar trend of lower energy intake was observed in the other BMI groups as well, although non-significant. No statistical evidence was found for differences in emotional eating between control group and intervention group participants at any of the study measurement points, neither could a clear trend be distinguished (Table 2).

At six months follow-up, restrained eating did no longer significantly differ between control group and intervention group, although the difference within the normal weight stratum remained (1.3 points higher in the intervention group; 95% CI 0.5, 2.1; *p =* 0.001). The difference in uncontrolled eating score also faded at six months follow-up. However, within the overweight BMI stratum, the intervention group had 2.2 points higher uncontrolled eating scores compared to the control group (95% CI 0.7, 3.7; *p =* 0.006). In the normal weight and obese BMI groups, a trend was observed of lower uncontrolled eating scores in the intervention group compared to the control group, yet not statistically significant. Energy intake remained 138 kcal lower in the obese stratum of the intervention group, although the difference with the control group was no longer significant (*p =* 0.09) (Table 2).

### 3.2. Physical Activity and Sedentary Time

Women with overweight in the intervention group had a borderline non-significantly higher physical activity compared to women with overweight in the control group at follow-up (log of MET-minutes 0.265; 95% CI −0.001, 0.531; *p* = 0.053). Furthermore, physical activity was generally higher among the intervention group compared to the control group, yet non-significant. Differences were especially large in the subgroup with obesity at end of intervention (3032 MET-minutes per week; 95% CI 2070, 4867 in the intervention group compared to 2491; 95% CI 1167, 5315 in the control group, *p* = 0.30) and at follow-up (3236 MET-minutes per week; 95% CI 1789, 5412 in the intervention group compared to 2255; 95% CI 960–4576 in the control group, *p* = 0.06) (Table 2). No statistical evidence was found for differences in sedentary time between control group and intervention group participants at any of the study measurement points, although a trend was observed of lower sedentary time in the intervention group compared to the control group (Table 2).

## 4. Discussion

This paper reports on the first e-health supported randomized controlled trial (RCT) focusing on lifestyle behavior in postpartum women with preceding excessive gestational weight gain. We show that the INTER-ACT postpartum lifestyle intervention is effective in enhancing restrained eating and in reducing both uncontrolled eating and energy intake in this population of women. These changing behaviors are important steps towards promoting a healthy postpartum lifestyle in this high-risk group. A healthier lifestyle has the potential to contribute to better long-term outcomes such as reduced obesity and better pregnancy- and birth related outcomes in a next pregnancy [3,6,8]. The intervention did not significantly affect emotional eating, physical activity and sedentary time.

There is dissent on whether or not an increased restrained eating score is favorable and should be encouraged. On the one hand it is argued that restrained eating contributes to weight loss, potentially mediated by decreased energy intake [3,20,21,22,23,24]. On the contrary, restrained eating is also thought to induce episodes of uncontrolled eating, which could nullify the beneficial effects on energy intake or potentially even result in an increased energy intake [25]. However, it is unlikely that the latter situation applies in the current study population, since simultaneously with the elevated restrained eating score, lower uncontrolled eating scores and energy intake were observed in the intervention group compared to the controls.

Remarkably, our results also revealed that at six months follow-up, uncontrolled eating was higher among intervention participants with overweight compared to controls with overweight, even though scores did not differ at the end of the intervention. This might signify that once the lifestyle intervention is finished, there is a feeling of letting loose that causes a relapse towards old behaviors.

No statistical evidence was found for an intervention effect on physical activity and sedentary time. However, generally a trend was observed of higher physical activity levels in the intervention group compared to controls, yet it was not statistically significant. Previous postpartum interventions did result in significantly increased physical activity [26,27], although not all [28]. Notably, the latter study was a combined lifestyle intervention focusing on both physical activity and nutrition, alike our intervention, whereas the interventions that did effectively increase physical activity had a focus on physical activity alone. This might potentially indicate that combined lifestyle interventions are not the most effective approach when increasing physical activity is the aim. This should however be further investigated in for example a meta-analysis. Alike physical activity, neither did sedentary time significantly improve in the intervention group compared to controls. This is not surprising due to the early postpartum being principally characterized by sedentariness inherent to infant feeding.

Overall, the current study showed effectiveness, especially in the normal weight subgroup. This is an important finding, as women who had an initial normal weight BMI are prone to shifting to the overweight BMI category between pregnancies [29]. Increasing interpregnancy BMI increases the risk of pregnancy- and birth-related complications in the next pregnancy such as gestational diabetes, pregnancy-induced hypertension, cesarean section, and macrosomia [29]. Improving lifestyle in the postpartum period might prevent this shift to a higher BMI and consequently decrease the risk of pregnancy- and birth related complications in a next pregnancy. A reduction of energy intake that is sustained for a longer period of time can contribute to weight loss and therewith a decreased postpartum weight retention. As the difference in energy intake between intervention and control group in the current study faded at six months follow-up, the intervention might potentially not have sustained long enough to lead to less postpartum weight retention in the intervention group. Future analyses will point out whether the INTER-ACT intervention affects postpartum weight retention after intervention and at follow-up.

Simultaneously, the largest effect sizes for energy intake and physical activity were observed among women with a BMI in the obese range. Physical activity at follow-up was even approximately 40% higher in the intervention group compared to controls. The differences between intervention and control groups were however in most cases not statistically significant, which is probably a result of the relatively small sample size of this BMI stratum. The large effect sizes might indicate that the intervention is in fact appropriate for this subgroup or that women with an obese pre-pregnancy BMI are especially motivated to improve their lifestyle habits in postpartum. This is a potentially important finding since women with obesity are especially at risk of long-term morbidity and passing on an increased risk of obesity to their offspring.

This study is subject to limitations. Firstly, the measurement of the intervention effect coincided with the last coaching session of the intervention group. Consequently, the effects shown here might not represent the full effects, as the effect obtained through this last coaching visit is not captured in the measurement. This might imply that intervention effects were potentially larger in reality, and not fully represented by the current figures.

Secondly, we found a large variation in the App use in the intervention arm: 11% did not use the App at all. If we look at the activity percentage, defined as number of days the app was used, divided by the total number of days between the week 6 and month 6 measurements (i.e., the duration of the intervention), and compare passive to active users (according to their activity percentage), we found that App use was related to the highest degree of education (higher use in university) and composition of the family (higher use in one-parent families). Furthermore, there was no statistical evidence of the activity percentage with ethnicity, employment status, income, number of hours sleep, sleep problems, or breast feeding the baby. Thirdly, the mean energy intake values are very low in both intervention and control group, and across BMI categories. This is likely due to underreporting, which is not uncommon in food frequency questionnaires [30]. Park et al. [30] concluded that average underreporting of absolute energy intake by the Food Frequency Questionnaire (FFQ) compared with an objectively measured energy biomarker, ranged from 25% to 40% and underreporting was more prevalent among obese individuals. This explains the range of a 25% to 40% lower energy intake level as mentioned here, between 1100 to 1400 kcal. As FFQ provides important information about episodically consumed foods, and assuming that the underreporting is systematic, we think that these values do not jeopardize the comparability between intervention and control group, which was the main aim of this paper. However, it might have compromised generalizability and comparability of this study with other studies.

## 5. Conclusions

The INTER-ACT lifestyle intervention combines face-to-face coaching with a smartphone application, targeting women with excessive gestational weight gain in the preceding pregnancy. The current analyses revealed that the postpartum phase of this RCT was effective in improving nutrition-related outcomes but not physical activity-related outcomes. The improvements could not be sustained at six months follow-up, and therefore the long-term benefits of the intervention are uncertain.

## Figures and Tables

**Table 1:** Characteristics of the INTER-ACT participants.

	Overall *N* = 1046	Control Group *N* = 504	Intervention Group *N* = 542
**Age**, mean (SD)	31.3 (3.9)	31.4 (3.8)	31.2 (3.9)
**Highest education**, *n* (percent)			
Up to secondary education	296 (28.4)	144 (28.8)	152 (28.1)
College	405 (38.9)	196 (39.2)	209 (38.6)
University	340 (32.7)	160 (32)	180 (33.3)
Missing	5	4	1
**Parity**, *n* (percent)			
Primiparous	577 (55.2)	286 (56.7)	291 (53.7)
Multiparous	469 (44.8)	218 (43.3)	251 (46.3)
**Sex of infant**, *n* (percent)			
Boy	554 (53)	263 (52.2)	291 (53.7)
Girl	492 (47)	241 (47.8)	251 (46.3)
**Method of conception**, *n* (percent) ^a^			
Spontaneous	918 (90.1)	455 (92.1)	463 (88.2)
Assisted reproductive technology	101 (9.9)	39 (7.9)	62 (11.8)
Missing	27	10	17
**Pre-pregnancy BMI category**, *n* (percent)			
Normal weight	509 (48.7)	249 (49.4)	260 (48)
Overweight	374 (35.8)	181 (35.9)	193 (35.6)
Obesity	163 (15.6)	74 (14.7)	89 (16.4)
**Gestational weight gain in kg**, median (IQR)			
Among normal weight ^b^	18.5 (17–20.9)	19 (17–21)	18 (17–20)
Among overweight	16 (13.5–19)	16 (13–18.9)	16 (14–19)
Among obese	14 (12–17)	14 (12–17)	14 (11.3–17)

^a^ Statistically significant difference between control and intervention group (*p* = 0.04). ^b^ Statistically significant difference between control and intervention group (*p* = 0.03).

**Table 2:** Univariate and multivariate analyses of the effect of the INTER-ACT randomized controlled trial on lifestyle behaviors.

Outcome	Time Point	BMI Group ^a^	Univariate Analyses					Multivariate Analyses		
			Control Group		Intervention Group		*p*-Value ^b^	*n*		*p*-Value ^c^
			*n*	Mean (SD), Median (Q1–Q3) or *n* (%)	*n*	Mean (SD), Median (Q1–Q3) or *n* (%)			Mean Difference (95% CI) or OR (95% CI)	
Restrained eating	Baseline (6 weeks PP)	Overall	513	13.4 (3.4)	550	13.3 (3.3)				
		NW	252	13.6 (3.6)	259	13.5 (3.3)				
		OW	188	13.3 (3.1)	200	13.1 (3.3)				
		OB	73	13.3 (3.2)	91	13.3 (3.1)				
	End of intervention (6 months PP)	Overall	377	14.3 (3.5)	374	15.3 (3.5)	**<0.001**	729	1.0 (0.5 to 1.5)	**<0.001**
		NW	199	14.1 (3.8)	187	15.4 (3.7)	**0.001**	375	1.4 (0.6 to 2.1)	**<0.001**
		OW	125	14.7 (3.1)	137	15.2 (3.5)	0.19	253	0.5 (−0.3 to 1.3)	0.23
		OB	53	14.1 (3.3)	50	15.2 (3.3)	0.08	96	0.4 (−0.8 to 1.6)	0.54
	6-month follow-up (12 months PP)	Overall	285	14.5 (3.6)	276	14.8 (3.2)	0.19	558	0.5 (−0.1 to 1.1)	0.10
		NW	148	14.0 (3.7)	133	14.9 (3.2)	**0.04**	268	1.3 (0.5 to 2.1)	**0.001**
		OW	99	14.9 (3.2)	110	14.7 (3.3)	0.77	203	0.0 (−0.9 to 0.9)	0.97
		OB	38	15.3 (4.1)	33	15.2 (3.3)	0.85	68	−0.3 (−2.1 to 1.5)	0.73
Uncontrolled eating	Baseline (6 weeks PP)	Overall	513	20.2 (5.5)	550	20.4 (5.5)				
		NW	252	19.5 (5.4)	259	19.1 (5.1)				
		OW	188	20.3 (5.4)	200	21.6 (5.6)				
		OB	73	22.5 (5.6)	91	21.7 (5.5)				
	End of intervention (6 months PP)	Overall	377	20.6 (5.9)	374	19.6 (5.6)	**0.02**	729	−1.0 (−1.9 to −0.2)	**0.02**
		NW	199	19.8 (5.7)	187	18.1 (5.1)	**0.003**	375	−1.6 (−2.7 to −0.5)	**0.004**
		OW	125	20.6 (5.7)	137	20.9 (5.7)	0.67	253	0.13 (−1.3 to 1.6)	0.86 ^d^
		OB	53	23.8 (6.1)	50	21.8 (5.8)	0.09	101	−1.6 (−4.0 to 0.8)	0.19
	6-month follow-up (12 months PP)	Overall	286	19.7 (5.6)	276	19.7 (5.7)	0.88	559	0.0 (−0.9 to 0.9)	0.85
		NW	149	19.0 (5.2)	133	18.1 (5.2)	0.18	278	−1.0 (−2.2 to 0.2)	0.09
		OW	99	19.4 (5.3)	110	21.3 (6.1)	**0.02**	194	2.2 (0.7 to 3.7)	**0.006** ^d^
		OB	38	23.0 (6.6)	33	21.0 (5.3)	0.18	71	−2.1 (−5.0 to 0.9)	0.18
Emotional eating, continuous	Baseline (6 weeks PP)	Overall	513	6.0 (4.0–8.0)	550	6.0 (4.0–9.0)				
		NW	252	5.0 (3.0–8.0)	259	5.0 (3.0–8.0)				
		OW	188	6.0 (5.0–9.0)	200	7.0 (5.0–9.0)				
		OB	73	8.0 (5.5–9.5)	91	8.0 (6.0–10.0)				
	End of intervention (6 months PP)	Overall	377	6.0 (4.0–9.0)	374	6.0 (4.0–9.0)	0.88			
		NW	199	5.0 (3.0–8.0)	187	5.0 (3.0–8.0)	0.83			
		OW	125	6.0 (4.5–9.0)	137	7.0 (4.0–9.0)	0.82			
		OB	53	9.0 (5.0–10.5)	50	8.0 (6.0–10.0)	0.93			
	6-month follow-up (12 months PP)	Overall	286	6.0 (4.0–8.0)	276	6.0 (4.0–9.0)	0.26			
		NW	149	6.0 (4.0–7.0)	133	5.0 (3.0–8.0)	0.50			
		OW	99	7.0 (4.0–9.0)	110	7.0 (5.0–10.0)	0.07			
		OB	38	8.0 (5.0–11.0)	33	8.0 (5.5–9.5)	0.90			
High emotional eating (score > 6)	Baseline (6 weeks PP)	Overall	513	221 (43.1%)	550	264 (48.0%)				
		NW	252	84 (33.3%)	259	89 (34.4%)				
		OW	188	90 (47.9%)	200	115 (57.5%)				
		OB	73	47 (64.4%)	91	60 (65.9%)				
	End of intervention (6 months PP)	Overall	377	165 (43.8%)	374	169 (45.2%)	0.70	729	1.04 (0.78 to 1.40)	0.78
		NW	199	68 (34.2%)	187	63 (33.7%)	0.92	375	0.94 (0.61 to 1.45)	0.79
		OW	125	59 (47.2%)	137	73 (53.3%)	0.33	253	1.21 (0.73 to 1.99)	0.46 ^e^
		OB	53	38 (71.7%)	50	33 (66%)	0.53	101	0.80 (0.34 to 1.87)	0.60
	6-month follow-up (12 months PP)	Overall	286	128 (44.8%)	276	131 (47.5%)	0.52	559	1.12 (0.80 to 1.56)	0.52
		NW	149	54 (36.2%)	133	46 (34.6%)	0.77	285	0.93 (0.56 to 1.52)	0.76
		OW	99	50 (50.5%)	110	61 (55.5%)	0.47	203	1.18 (0.67 to 2.05)	0.57 ^e^
		OB	38	24 (63.2%)	33	24 (72.7%)	0.39	71	1.44 (0.51 to 4.05)	0.49
Energy intake (kcal)	Baseline (6 weeks PP)	Overall	517	1399 (378)	553	1409 (414)				
		NW	254	1351 (344)	262	1398 (430)				
		OW	189	1443 (414)	200	1390 (356)				
		OB	74	1448 (376)	91	1482 (475)				
	End of intervention (6 months PP)	Overall	378	1277 (375)	377	1204 (364)	**0.006**	732	−69 (−123 to −15)	**0.01**
		NW	199	1249 (381)	189	1199 (390)	0.20	377	−43 (−121 to 36)	0.30
		OW	126	1277 (361)	138	1201 (325)	0.07	254	−75 (−161 to 11)	0.09
		OB	53	1385 (372)	50	1230 (372)	**0.04**	94	−174 (−314 to −35)	**0.01**
	6-month follow-up (12 months PP)	Overall	286	1174 (339)	279	1172 (333)	0.95	562	−1 (−62 to 59)	0.96
		NW	149	1139 (324)	135	1146 (313)	0.85	287	15 (−70 to 101)	0.74
		OW	99	1208 (361)	110	1224 (343)	0.75	199	28 (−68 to 125)	0.57
		OB	38	1220 (328)	34	1106 (361)	0.17	69	−138 (−296 to 20)	0.09
Physical activity (MET-minutes per week)	Baseline (6 weeks PP)	Overall	487	1857 (891–3438)	533	1728 (920–3279)				
		NW	236	1785 (892–3525)	252	1685 (787–3070)				
		OW	182	1924 (918–3030)	191	1837 (1118–3143)				
		OB	69	1860 (848–3725)	90	1899 (836–3673)				
	End of intervention (6 months PP)	Overall	344	2461 (1342–4691)	340	2640 (1372–5397)	0.40	649	0.052 (−0.099 to 0.203) ^f^	0.50
		NW	182	2406 (1372–4595)	168	2634 (1325–5294)	0.65	333	0.062 (−0.145 to 0.270) ^f^	0.56
		OW	114	2681 (1319–4593)	131	2547 (1299–5876)	0.98	230	−0.036 (−0.296 to 0.223) ^f^	0.78
		OB	48	2491 (1167–5315)	41	3032 (2070–4867)	0.30	75	0.104 (−0.292 to 0.501) ^f^	0.60
	6-month follow-up (12 months PP)	Overall	269	2403 (1240–4543)	261	2814 (1501–5312)	0.11	497	0.144 (−0.025 to 0.313) ^f^	0.09
		NW	143	2616 (1107–4650)	128	2519 (1392–4103)	0.11	251	−0.018 (−0.256 to 0.220) ^f^	0.88
		OW	94	2132 (1417–4072)	101	3210 (1530–6293)	0.70	181	0.265 (−0.001 to 0.531) ^f^	**0.053**
		OB	32	2255 (860–4576)	32	3236 (1789–5412)	0.06	61	0.413 (−0.128 to 0.953) ^f^	0.14
Sedentary time (minutes per day)	Baseline (6 weeks PP)	Overall	486	325 (171)	532	323 (180)				
		NW	235	325 (183)	252	313 (171)				
		OW	182	324 (151)	191	332 (193)				
		OB	69	326 (180)	89	330 (178)				
	End of intervention (6 months PP)	Overall	343	308 (167)	338	293 (162)	0.21	661	−14 (−39 to 12)	0.30
		NW	182	319 (161)	167	299 (170)	0.26	339	−21 (−57 to 15)	0.26
		OW	113	310 (179)	130	286 (163)	0.28	232	−24 (−65 to 18)	0.29
		OB	48	266 (160)	41	288 (120)	0.46	81	31 (−28 to 90)	0.30
	6-month follow-up (12 months PP)	Overall	269	317 (160)	261	299 (173)	0.22	511	−17 (−46 to 13)	0.28
		NW	143	319 (158)	128	287 (155)	0.09	263	−33 (−72 to 6)	0.12
		OW	94	311 (157)	101	302 (182)	0.74	178	−17 (−61 to 26)	0.43
		OB	32	321 (176)	32	334 (213)	0.79	58	−3 (−90 to 85)	0.95

Abbreviations: MET = metabolic equivalent of task; NW = normal weight; OW = overweight; OB = obesity; PP = postpartum. ^a^ Pre-pregnancy BMI category. ^b^ *p*-value for differences between control group and intervention group, using independent samples *t*-test, Mann-Whitney U test, or likelihood ratio test. ^c^ *p*-value for difference between groups, adjusted for method of conception and kilograms of excessive weight gain, using multivariate linear or logistic regression. ^d^ Post-hoc analyses with baseline uncontrolled eating scores added to the model (due to significant difference at baseline, *p* = 0.02) did not change the results (data not shown). ^e^ Post-hoc analyses with baseline percentage of women with high emotional eating score added to the model (due to significant difference at baseline, *p* = 0.05) did not change the results (data not shown). ^f^ Log of MET-minutes. *p*-Values < 0.05 are indicated in bold.
